# Nitric Oxide Modulates Metabolic Processes in the Tumor Immune Microenvironment

**DOI:** 10.3390/ijms22137068

**Published:** 2021-06-30

**Authors:** Christopher L. McGinity, Erika M. Palmieri, Veena Somasundaram, Dibyangana D. Bhattacharyya, Lisa A. Ridnour, Robert Y. S. Cheng, Aideen E. Ryan, Sharon A. Glynn, Douglas D. Thomas, Katrina M. Miranda, Stephen K. Anderson, Stephen J. Lockett, Daniel W. McVicar, David A. Wink

**Affiliations:** 1 Laboratory of Cancer ImmunoMetabolism, Center for Cancer Research, National Cancer Institute, National Institutes of Health, Frederick, MD 21702, USA; chris.mcginity@nih.gov (C.L.M.); erikamariana.palmieri@nih.gov (E.M.P.); veenas10@gmail.com (V.S.); dana.bhattacharyya@nih.gov (D.D.B.); ridnourl@mail.nih.gov (L.A.R.); robert.cheng2@nih.gov (R.Y.S.C.); andersonst@mail.nih.gov (S.K.A.); McvicarD@mail.nih.gov (D.W.M.); 2 Lambe Institute for Translational Research, School of Medicine, National University of Ireland Galway, H91 TK33 Galway, Ireland; aideen.ryan@nuigalway.ie (A.E.R.); sharon.glynn@nuigalway.ie (S.A.G.); 3 Department of Medicinal Chemistry and Pharmacognosy, College of Pharmacy, University of Illinois at Chicago, Chicago, IL 60607, USA; ddthomas@uic.edu; 4 Department of Chemistry, University of Arizona, Tucson, AZ 85721, USA; kmiranda@email.arizona.edu; 5 Optical Microscopy and Analysis Laboratory, LEIDO Biomedical Research Inc., Frederick National Laboratory for Cancer Research, Frederick, MD 21702, USA; locketts@mail.nih.gov

**Keywords:** cancer, immunometabolism, biochemistry

## Abstract

The metabolic requirements and functions of cancer and normal tissues are vastly different. Due to the rapid growth of cancer cells in the tumor microenvironment, distorted vasculature is commonly observed, which creates harsh environments that require rigorous and constantly evolving cellular adaption. A common hallmark of aggressive and therapeutically resistant tumors is hypoxia and hypoxia-induced stress markers. However, recent studies have identified alterations in a wide spectrum of metabolic pathways that dictate tumor behavior and response to therapy. Accordingly, it is becoming clear that metabolic processes are not uniform throughout the tumor microenvironment. Metabolic processes differ and are cell type specific where various factors promote metabolic heterogeneity within the tumor microenvironment. Furthermore, within the tumor, these metabolically distinct cell types can organize to form cellular neighborhoods that serve to establish a pro-tumor milieu in which distant and spatially distinct cellular neighborhoods can communicate via signaling metabolites from stroma, immune and tumor cells. In this review, we will discuss how biochemical interactions of various metabolic pathways influence cancer and immune microenvironments, as well as associated mechanisms that lead to good or poor clinical outcomes.

## 1. Introduction

Prognosis and treatment of many cancers is largely impacted by cancer and stromal cell communication that shapes the tumor microenvironment (TME). The TME is an interactive landscape where cancer cells do not act in isolation, but rather, in combination with other host cells, including stromal and immune cells. Together, these components form unique heterogenous cellular neighborhoods within the tumor, serving as relays that receive, process, and transmit tumor status from one neighborhood, or location, to other distant neighborhoods. In concert, these neighborhoods work together to serve as the *structural mediators* for cancer and aid to promote an aggressive and therapeutically resistant cancer type.

While there are subtle differences between phenotypes, cancer cells generally fall into two main categories: (1) proliferative and (2) cancer stem cell (CSC)-like, the latter of which has a spectrum of phenotypes from mesenchymal stem cell-like (i.e., MDA-MB-231 triple negative breast cancer cell line) to embryonic stem cell-like cells [1,2]. Proliferative cancer cells are mostly epithelial-like, and their function is to convert nutrients into tumor mass, resulting in tumor proliferation. CSC-like cancer cells can remain dormant and resistant to treatment or self-renew and differentiate into a number of cell types allowing them to seed new tumor sites upon activation [3]. Thus, CSC-like cancer cells are thought to determine cancer aggressiveness by promoting chemoresistance, immunosuppression, and metastasis. On the other hand, non-cancer cells, or tumor-associated stromal cells (TASCs), consist of several cell types including fibroblasts, immune and vascular cells, and in some cases, neurons. The main function for TASCs is to provide support to the primary tumor by supplying essential nutrients and facilitating matrix remodeling that can support tumor implantation and promote metastasis. Importantly, these support cells can originate from tumor and/or host stromal and immune cells.

Metastasis and resistance to treatment, including innate chemoresistance of cancer cells and immune polarization, are key factors that determine poor prognosis, and both are dependent on the above-mentioned cancer-TASC cell interactions. For example, metastatic cancer cells are impacted by a host of stromal factors including matrix metalloproteins (MMPs), which promote extracellular matrix (ECM) remodeling, as well as cytokines, which increase cancer cell epithelial to mesenchymal transition (EMT) that when combined, ultimately lead to metastasis. Thus, the success of cancer treatment, including conventional surgery, radiation, and chemotherapy, is highly dependent on the polarization of the immune system.

The notion of communication through intercellular signaling has classically involved structure-function interactions. In addition, redox regulated signaling has provided new strategies to enhance cancer therapy that have included modification of signal transduction, immune signaling, inhibition of nitric oxide synthase 2 (NOS2), and cyclooxygenase 2 (COX2), which have all shown promise in preclinical and even clinical trials [4,5,6]. Furthermore, NOS2/COX2 are linked to poor outcome and collaborate in feedforward loops in both cancer and immune cells in several tumor types, with ER- breast cancer being the best example [7,8,9,10,11,12,13,14,15,16,17,18]. Importantly, the collaboration of these two enzymes involving an array of oncogenic signaling mechanisms in the promotion of disease progression was recently reported [7].

In addition to oncogenic signaling mechanisms, NOS2-derived NO can also affect and tune anabolic and catabolic metabolism, including sugar, fatty acid, and amino acid metabolism, which are now considered as novel areas of cancer treatment research for the most clinically challenging cancers. Importantly, these are the functional mediators that carry messages to and from various tumor neighborhoods to impact patient outcome. Thus, developing novel treatments from the perspective of metabolism may also offer unique targets in addition to the signal transduction inhibitors and immunotherapy that improve the efficacy of current cancer therapies. In this review, we will identify key metabolites that serve as both intra- and inter-cellular signaling molecules to tune local and distant sites of the TME. Accordingly, we will also describe mechanisms for how the interaction of these metabolic pathways may set conditions that can be used as markers to determine patient outcome and provide further targets for cancer treatment.

The investigation of metabolism in the context of cancer and NO can be extremely complex, because metabolic and NO redox mechanisms are not static. In contrast, these mechanisms change dynamically in adaptive tumor environments. Critical metabolic switches that not only affect global metabolism within the cell also impact adjacent and distal cells of various phenotypes across different spatial and temporal domains, which makes metabolism research a daunting endeavor. Therefore, approaching cancer metabolism from a systems point of view to integrate different pathways into a description of the entire cancer cell population as a whole is necessary. For this purpose, the biochemical pathways of interest may first be discussed in isolation, which then can be grouped and placed within the context of their intersections that are critical to cancer metabolism. These nodes often have competing pathways that change the carbon flow depending on surrounding circumstances.

**Figure 1:**
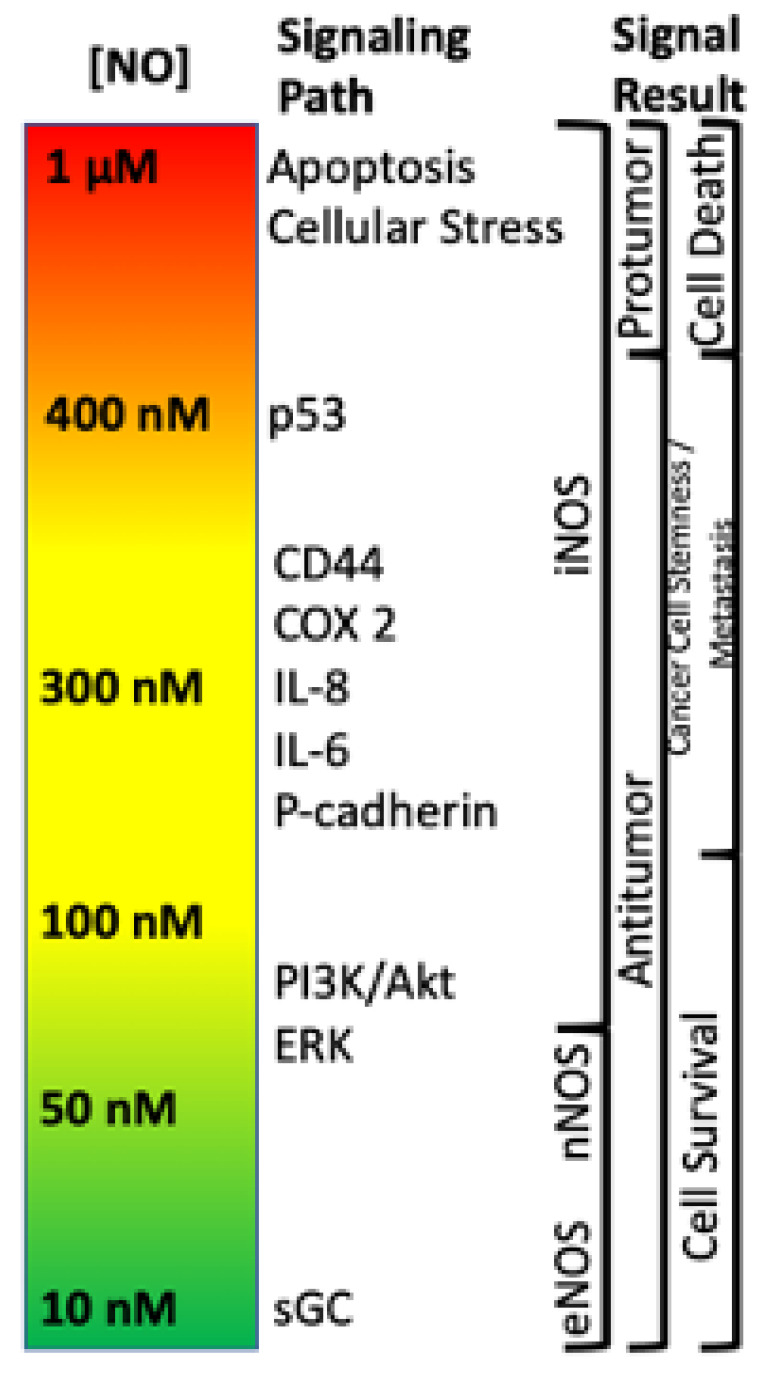
Nitric oxide concentration-dependent signaling and functional effects.

Within the TME, NO has been shown to drive various pro- and antitumor mechanisms that can lead to different disease outcomes. One of the most important findings is that NO and RNS progenitor signaling is a function of NO concentration, and different concentrations drive distinct signaling cascades. For example, it has previously been shown that NO levels which describe pro- and antitumor activity (Figure 1) can be divided into three main categories: (1) cGMP signaling (<100 nM NO), (2) pro-oncogenic nitrosative signaling (100–500 nM NO), and (3) nitrosative stress signaling (500–2000 nM NO) [19,20,21]. However, while classical oncogenic signaling has been described with respect to NO concentration, no description of the impact of NO/RNS biochemistry on the metabolic pathways that tune the TME exist. Here, we will examine literature relating to the three basic levels of NO signaling to discuss critical metabolic nodes in cancer and how these nodes may influence the TME and overall clinical outcomes.

## 2. The Master Nodes

Cancer metabolism starts with pathways associated with fatty acids and sugars (CHO), with the tricarboxylic acid cycle (TCA), oxidative phosphorylation (OxPhos), glycolysis and fatty acid oxidation (FAO) being the hubs of interaction for these metabolic paths. Accordingly, the differential utility for these macronutrients in tumor and immune cells has been described, showing that cell types utilize these same metabolic paths, but for different functional purposes. For example, many have defined an aggressive cancer in terms of the ‘Warburg Effect’, in which cancer cells, heavily rely on aerobic glycolysis for rapid proliferation, in addition to OxPhos. On the other hand, activation of immune cells, including dendritic cells (DCs), macrophage and T cells also prompts the acquisition of a metabolic status highly dependent on aerobic glycolysis for sustained production of proinflammatory cytokines and host defense mechanisms. Alternatively, quiescent, and non-stimulated immune cells primarily utilize OxPhos for energy production, indicating increased reliance on OxPhos over glycolysis is necessary for wound healing and an immunosuppressive response in immune cells. Unfortunately, the requirement for OxPhos and glycolysis by both cancer and immune cells, also creates a hostile environment in which tumor and immune cells compete for the same nutrients to support their functions. This often results in the stripping of nutrients by the tumor from the host immune system, leading to immune evasion as well as poor prognosis and disease outcomes.

### 2.1. G6P

Glucose utilization in glycolysis results in the production of pyruvate for TCA use and further continuation to ATP production via cellular respiration. However, an alternative glucose utilization path involves its diversion from glycolysis to the pentose phosphate pathway (PPP) via glucose-6-phosphate (G6P), thus decreasing the glycolysis-derived pyruvate supply for the TCA. Whereas G6P use for glycolysis is viewed as a catabolic process, G6P use by the PPP is anabolic and supports the building of new biomolecules such as ribose. PPP use of G6P results in the production of NADPH during the oxidative phase. Conversely, during the non-oxidative phase G6P is used for constructing the cellular building blocks ribose-5-phosphate (R5P) for nucleotide assembly and erythrose-4-phosphate (E4P) for aromatic amino acid synthesis [22]. Thus, G6P is a critical metabolic node that connects cellular catabolism through glycolysis and anabolism through the PPP.

In addition to an upregulation in glycolytic flux, flow through specific points of the PPP is also increased in several cancer types and this is thought to support anabolic processes associated with tumor proliferation [23,24]. Alternatively, immune cell activation also stimulates carbon flow through the PPP (as well as glycolysis) to support NADPH production for NADPH oxidase, NO synthase (NOS) and other antioxidant pathways such as thioredoxin (Trx)—all of which are used for host defense [25,26].

### 2.2. Pyruvate

**Figure 2:**
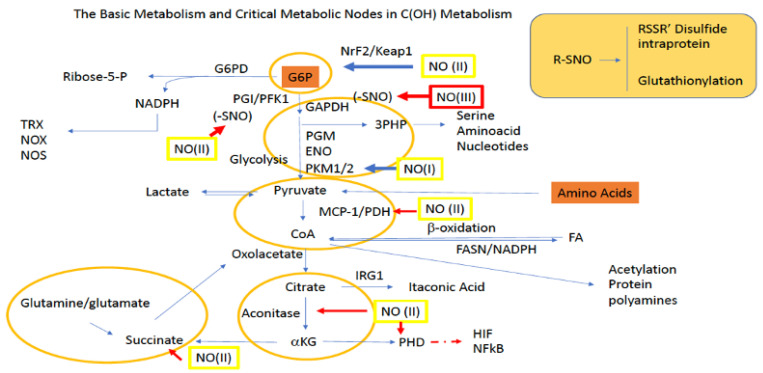
Sites for NO activity along glycolysis, the PPP, and the TCA. Green boxes/(II) represent intermediate levels of NO (nitrosative signaling) and red boxes/(III) indicate high levels of NO (nitrosative stress) required for NO interaction at the indicated site. Red arrows indicate inhibitory mechanisms while blue arrows indicate activating mechanisms.

The final step of glycolysis involves the conversion of phosphoenolpyruvate (PEP) to pyruvate by PKM1/2. Additionally, pyruvate can be made in a glycolysis-independent manner via the metabolism of amino acids, which include alanine, serine, and methionine, as well as some of their other derived species. Once made, pyruvate can enter the mitochondria through mitochondrial pyruvate carriers MPC1 and MPC2. In the mitochondria, pyruvate donates its acetyl functional group to coenzyme A (CoA) via pyruvate dehydrogenase (PDH), forming acetyl-CoA. Acetyl-CoA then undergoes condensation with oxaloacetate (OAA), which is facilitated by citrate synthase (CS), and this allows the resulting product citrate to enter the TCA for additional energy production. However, during TCA blockade (examples described below), pyruvate can also directly stimulate TCA cycling via its conversion to OAA by pyruvate carboxylase (PC). Additionally, pyruvate derived OAA may also be utilized for the production of glucose through G6P in gluconeogenesis (Figure 2).

Yet another fate for pyruvate is its diversion from the TCA cycle altogether via its reduction to lactate by lactate dehydrogenase (LDH). From here lactate, which can be used within the cell and/or transported by monocarboxylate transporters (MCTs) to other cells, can be converted back to pyruvate in the liver to participate in the Cori cycle and/or gluconeogenesis for glucose production. Importantly, the ability of lactate to be transported amongst cells allows it to serve as a critical redox biochemistry signaling mediator for reporting global glycolytic status. Altogether, pyruvate connects key metabolic pathways including glycolysis, the TCA cycle, gluconeogenesis, and lactate metabolism (Figure 2).

### 2.3. Ketoglutarate Node

*a*-KG is formed as a TCA intermediate from the metabolism of isocitrate by isocitrate dehydrogenase (IDH) [27]. Thus, limiting levels of isocitrate prevents *a*-KG biosynthesis. *a*-KG sits at a critical node in cancer cell metabolism by providing flexibility between facilitating forward or reverse TCA cycling. Forward TCA metabolism of *a*-KG occurs via *a*-KG dehydrogenase (α-KGDC), which contains a dihydrolipoamide succinyltransferase (E1), (E2) and dihydrolipoamide dehydrogenase (E3) to irreversibly form succinyl-CoA [27]. Importantly, having the same cofactors as PDH, α-KGDH also has redox chemistry at its lipoate cofactor [28] similar to PDH [29]. Reverse TCA metabolism of *a*-KG involves reversible processing back to citrate, which can be used to generate CoA through lipogenesis, ultimately contributing to NADPH production for antioxidant and proliferative metabolism [30]. Importantly, only three irreversible TCA reactions exist: (1) citrate synthase (OAA + Acetyl-CoA → citrate); (2) IDH (isocitrate to αKG) and (3) α-KGDH (αKG to succinyl-CoA).

In the liver, *a*-KG can be diverted from the TCA to the Cahill cycle where it accepts an amino group from alanine to form glutamate and pyruvate through alanine transaminase (ALT). Alternatively, *a*-KG can also be exported from the mitochondria, and away from the TCA to the cytosol by the malate-*a*-KG antiporter to participate in the malate-aspartate shuttle. Notably, the ability of *a*-KG to be transaminated makes it paramount in regulating nitrogen toxicity. Moreover, transamination of *a*-KG followed by cellular export allows for intercellular nitrogen trafficking, making *a*-KG a critical redox biochemistry signaling mediator in reporting and regulating global nitrogen status.

### 2.4. Glutamine/Glutamate Node

**Figure 3:**
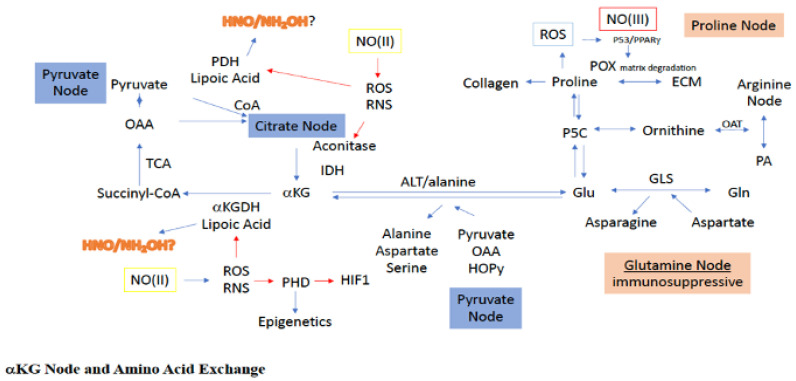
Sites for NO interaction with the a-KG node, as well as its associated amino acid paths. Green boxes/(II) represent intermediate levels of NO (nitrosative signaling) and red boxes/(III) indicate high levels of NO (nitrosative stress) required for NO interaction at the indicated site. Red arrows indicated inhibitory mechanisms while blue arrows indicate activating mechanisms.

One of the most important amino acids in cancer and immune cells, as well as other rapidly proliferating cells, is glutamine [31,32]. Glutamine is the most abundant amino acid in plasma and is essential for nourishing proliferating cells. In addition to being an energy source, glutamine, glutamate, and *a*-KG together form an intersection by which interconversion of many amino acids and metabolites can be facilitated [33,34]. Thus, accessibility to glutamine provides cancer cells with the flexibility to derive energy and various amino acids for anabolism under an otherwise nutrient deprived state. For example, during citrate blockade, either from redox species such as NO, ROS, and metal chelators (picolinic acid tryptophan metabolism), or diversion of glucose from the TCA cycle to lactate, glutamine provides an alternative fueling source, which serves as an additional mechanism to sustain respiration in some cells (Figure 3). Glutamine/glutamate provide an alternative mechanism to sustain mitochondrial respiration for both FA and glucose-derived acetyl-CoA [35]. Conversely, in immune cells, glutamine is also an important source of fuel and reflects the overall status of immune cells [36]. For example, inhibition of glutamine synthetase (GS) in macrophage converts these cells from an M2 to an M1 activated phenotype. This is achieved by decreasing intracellular glutamine, which increases succinate levels via an enhancement in glycolytic flux that both normalizes vasculature and limits metastasis [36]. Thus, metabolic tuning of the glutamate/glutamine axis has important effects on the disease progression of cancer.

During the deamidation of glutamate to *a*-KG, transfer of the glutamate-derived amino group can be used to construct a variety of amino acids, including alanine, aspartate, serine, glycine, isoleucine, leucine, valine, and ornithine [33]. An interesting example of this is the glutamate-dependent conversion of pyruvate to alanine and *a*-KG in the glucose-alanine cycle, which serves as a competitive pathway to the lactate and acetyl-CoA systems for pyruvate utilization (Figure 3). Indeed, pyruvate uptake and utilization for *a*-KG production has been witnessed during ECM remodeling of metastatic breast cancer cells [37]. More clearly, use of pyruvate for alanine synthesis limits pyruvate use for both lactate, via LDH, and acetyl-CoA, via PDH, production. Alanine is an intercellular trans metabolite, which when exported from organelles and cells, can be reconverted to pyruvate to produce glucose, making alanine important for energy balance.

Another important aspect of the glutamine node is the glutamate-dependent transamidation of OAA to aspartate. As previously stated, OAA is critical for the entrance of acetyl-CoA into the TCA, resulting in the formation of citrate. Thus, diversion of OAA through glutamate to aspartate leads to reduced OAA levels in the TCA, making OAA a sensor for metabolic status. Specifically, reduced OAA decreases acetyl-CoA conversion to citrate, essentially causing a blockade at the citrate node. However, such a citrate block can also be bypassed, since glutamate-dependent OAA conversion to aspartate yields *a*-KG as well, which can directly enter the TCA downstream from the citrate block (Figure 3). Thus, it seems more appropriate to consider the relationship between *a*-KG and OAA as describing the metabolic status for mitochondrial tuning of carbon flow. Altogether, within the inflammatory niche and tumor microenvironment, the glutamine/glutamate node is a critical link between amino acid metabolism and TCA carbon flow.

### 2.5. Proline Node

Under metabolically challenging conditions, as experienced in the tumor, proline and glycine are extracted from the ECM (Figure 3), which provides an important reservoir for these amino acids to serve in both anabolic and catabolic processes [38]. Proline can be exported from and taken up by other cells, as well as derived from autophagic digestion. In the cytosol, proline is generated from ornithine and *a*-KG via ornithine cyclodeaminase (OCD). Importantly, use of ornithine for proline biosynthesis serves to divert arginine metabolism from both polyamine synthesis and citrulline recycling to proline. Additionally, ornithine can be derived from the metabolism of glutamate through glutamate-5-semialdehyde (G5S), connecting proline metabolism to the glutamine/glutamate node as well, and making proline a potentially unique amino acid node for TCA rewiring through *a*-KG by decreasing glutamate levels. G5S is synthesized from glutamate by the aldehyde dehydrogenase 18A1 (ALDH18A1) and is in equilibrium with 1-pyrroline-5-carboxylate (P5C), the latter of which can be reduced by pyrolline-5-carboxylate reductase (PYCR) to proline [39]. Importantly, both ALDH18A1 and PYCR are controlled in luminal breast cancer by cMYC and PI3K [40]. Thus, proline biosynthesis and/or cellular consumption is critical for maintenance in a metabolically challenged cellular niche [41].

The first step in proline catabolism is oxidation to P5C by proline dehydrogenase/proline oxidase (PRODH/POX), which serves as a hub for proline metabolism (Figure 3). PRODH resides in the inner membrane of the mitochondria and generates FADH, which can enter the respiration chain leading to ATP [42]. However, higher levels of proline can lead to ROS generation through FADH_2_. Importantly, PRODH is induced by hypoxia and promotes pro-survival mechanisms, autophagy, and metastasis [43,44]. Another important pathway for proline consumption is its essential role in the formation of collagen and fibrosis, the latter of which is pertinent to the TME, which often displays extensive fibrosis. Notably, collagen formation is dependent on both matrix structure and PHD activity, which are O_2_, iron, *a*-KG, and ascorbic acid dependent. Thus, altogether, proline derived from the ECM and autophagy can serve as fuel, either directly or indirectly through glutamate/P5C, to maintain adequate *a*-KG levels and facilitate TCA cycling even under nutrient deprivation (Figure 3).

## 3. Fueling the Master Nodes

As alluded to above, a dichotomy exists in both cancer and immune cells for the utilization of glucose: glucose is needed to provide pyruvate and *a*-KG equivalents for energy production through the TCA and cellular respiration, and to facilitate G6P/PPP-dependent NADPH and macronutrient synthesis to support cellular redox and structural needs. Therefore, glucose utilization for one specific metabolic purpose will, at least partially, limit its availability for use in other pathways. To counter this, cancer employs multiple alternative metabolic pathways to achieve the same end product in what seems to be a redundant manner. However, the purpose for this is to simply prevent catastrophic loss of a certain metabolite when other metabolic paths pull this metabolite in other directions. As detailed above, glucose is one such metabolite, and therefore, its downstream master nodes G6P, pyruvate and *a*-KG must be maintained via both conventional and non-conventional biochemical metabolism.

In the following section, we will describe the key metabolic paths that the cell can choose from to give it the metabolic flexibility to maintain proper levels for the master node metabolites under varying conditions and environmental demands.

### 3.1. Direct Amino Acid Fueling

These metabolic nodes for amino acids are important in determining alternative fuel sources as well as providing necessary components for proliferation under harsh nutrient-poor conditions often found in tumors. With respect to the immune system, these amino acid pathways help facilitate one of the critical strategies for fighting pathogens and tumors, which is to deprive them of amino acids such as tryptophan and arginine. Cancer cells exposed to these undernourished conditions must utilize alternate available biochemistry to survive. For example, primary 4T1 tumors are glutaminase (GLS) sensitive, indicating their reliance on glutamine, while lung metastases are PC sensitive and thus, pyruvate/OAA dependent [45,46,47].

Direct amino acid fueling of the master nodes is done through many amino acids such as serine, threonine, glutamine, proline, aspartate, alanine, and cysteine. From the perspective of overall metabolism for cancer cells, amino acid fueling can be viewed as mechanisms of TCA anaplerosis and cataplerosis which means filling or emptying the pool. These terms mean that at critical junctures, or nodes, in the metabolic network, biochemical pathways can either increase or decrease these TCA pools of critical metabolites. With respect to amino acids, there are two major entry points for TCA anaplerosis, pyruvate and *a*-KG. These two nodes link multiple pathways that can interchange metabolic and amino acid products to provide necessary nutrient requirements under stressful conditions [33,48,49,50]. The reversibility of these systems offers maximal options for anabolic and catabolic processes in cellular niches and collaboration between cancer, immune and stromal cells.

**Figure 4:**
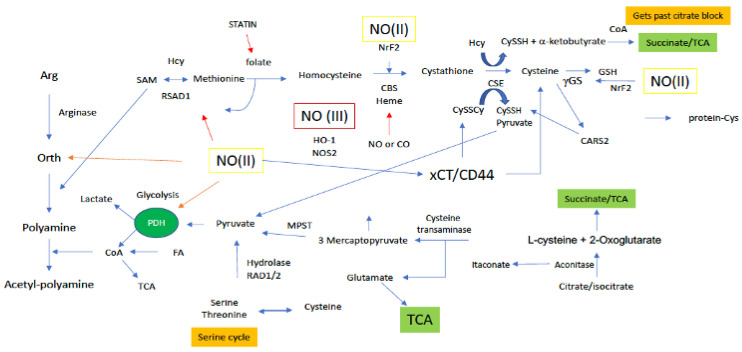
Sites along arginine and sulfur metabolic paths for NO interaction. Green boxes/(II) represent intermediate levels of NO (nitrosative signaling) and red boxes/(III) indicate high levels of NO (nitrosative stress) required for NO interaction at the indicated site. Red arrows indicated inhibitory mechanisms while blue arrows indicate activating mechanisms.

Pyruvate entry to the TCA either requires its carboxylation to OAA via PC (Figure 3), or conversion to acetyl-CoA via PDH (Figure 4). Conversely, TCA-dependent anaplerosis of the *a*-KG node occurs through citrate, isocitrate, aconitase and IDH, or through TCA-independent anaplerosis from ALT mediated glutamate deamidation. The latter provides an important intersection for a number of amino acid pathways with the TCA. For example, glutamine can be converted to glutamate via glutaminase, linking glutamine to the TCA as well [51]. Importantly, there are several inhibitors that target cancer cell growth, respiration and immunosuppressive mechanisms through glutamine lysis [33]. Indeed, there are also specific amino acids that feed the pyruvate node such as alanine, serine, threonine, glycine, and cysteine.

#### 3.1.1. Pyruvate

##### Aspartate

Aspartate is an important *transmetabolite* that utilizes the pyruvate node product OAA (Figure 3). In the malate-aspartate shuttle, mitochondrial aspartate resulting from the transamination of OAA by aspartate transaminase (AST), is exported to the cytosol via the glutamate-aspartate antiporter [52]. In the cytosol, aspartate then undergoes deamination by cytosolic AST, converting it back to cytosolic OAA that may then directly enter gluconeogenesis for G6P production. Aspartate can also be involved in purine and pyrimidine synthesis. Furthermore, aspartate is important in arginine recycling from citrulline as well as conversion to glutamate and asparagine to facilitate ammonia transfer.

##### Alanine

Another *transmetabolite* for pyruvate anaplerosis is alanine. Alanine can be consumed in the diet and enter the cell through ASC1. Cytosolic alanine can be deaminated to pyruvate via ALT (Figure 3) for gluconeogenic use [53]. For this, reverse ALT catalysis of alanine + *a*-KG yields pyruvate, which can subsequently be carboxylated by pyruvate carboxylase (PC) to enter the gluconeogenic pathway for G6P production. Thus, alternative alanine use competes with alanine-dependent gluconeogenesis. The flexibility of these transmetabolites provide important anabolic and catabolic plasticity to adapt to metabolic stress communicated within organelles and different cells.

##### Methionine/Cysteines

**Figure 5:**
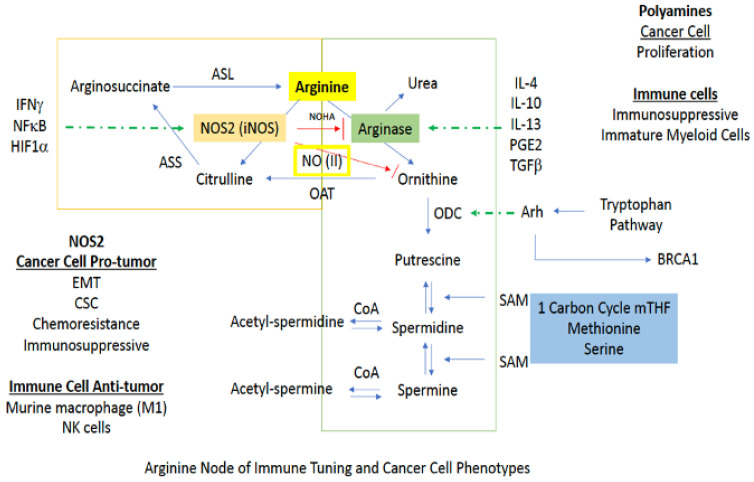
Downstream signaling pathways affected by NOS2/NO activity. Red arrows indicate inhibition while blue arrows indicated activation.

Methionine and its metabolism to other sulfur-based metabolites controls the greater part of the cellular thiol landscape. Biosynthesis of Met requires homocysteine (HCys) (Figure 4) and 5-methyltetrahydrofolate, which is derived from serine in the folate cycle. Met is the precursor for the primary methylating molecule SAM (Figure 5), which has roles in DNA methylation, energy production via creatine formation in the phosphagen cycle and, proliferation through polyamine synthesis. Thus, it appears these downstream metabolites are important cellular workhorses of Met-based metabolism, in addition to the central role of Met in protein synthesis. Met dependency is a phenomenon common to many cancer cell types that is characterized by the cells inability to grow in a medium supplemented with HCys in place of Met [54]. Interestingly, SAM supplementation of Met starved cancer cells was shown to counter Met dependency, suggesting that Met dependency may actually be better characterized as SAM dependency in certain cancers [55]. Cancer cells rely on polyamine production for a number of reasons, including facilitating cellular growth [56]. In this light, Met recruitment for polyamine production through SAM may also explain the Met dependency of certain cancer cells, not necessarily for Met, but rather, for polyamines. Lastly, cancer cells in general, including breast cancer, have elevated GSH levels, compared to their normal counterparts. Thus, to maintain high GSH levels, the cancer cell must also rely on a steady supply of the GSH precursor Cys, which is derived from Met via the transsulfuration path. Therefore, Met dependency may also be attributed to the cancer cells need to maintain high Cys production to sustain elevated GSH levels.

Cys enters the cell through the alanine/serine/Cys transporter ASC-1 to be utilized in the cytosol. Alternatively, biosynthesis of Cys occurs in the transsulfuration pathway from the amino acid serine and homocysteine (HCys; derived from Met), and during this process, pyruvate is also produced as a byproduct (Figure 4). Thus, transsulfuration-dependent pyruvate production is also largely reliant on Met and the Met cycle, and serine and the serine cycle. In the first step of the Met cycle, Met is methylated by methionine adenosyl synthetase (MAT) to *S*-adenosylmethionine (SAM) [57]. Methyl transfer from SAM ultimately results in the production of HCys, which can then enter the transsulfuration path. Canonical transsulfuration processing of HCys involves the two cytosolic enzymes cystathionine beta synthase (CBS) and cystathionine gamma lyase (CTH), which catalyze the conversion of HCys + serine to cystathionine, and cystathionine to Cys, respectively. Cytosolic Cys may then enter the sulfur transferase pathway where it is first deaminated to 3-mercaptopyruvate (3-MP) by cysteine aminotransferase (CAT) [58], followed by desulfuration via 3-mercaptopyruvate sulfertransferase (3-MPST), resulting in a 3-MPST bound hydropersulfide and pyruvate (Figure 4). Importantly, complete metabolism of Met, in combination with serine, through the Met cycle, transsulfuration and sulfur transferase paths affords two equivalents of pyruvate. However, Cys may also be diverted from the sulfur transferase path to be incorporated into proteins or glutathione (GSH). Use of Cys in this manner results in the production of only one equivalent of pyruvate from Met metabolism. Moreover, as mentioned above Met may be diverted from the transsulfuration and sulfur transferase paths altogether for polyamine synthesis. This completely pulls sulfur equivalents away from Cys production and results in no sulfur-dependent pyruvate production at all. Interestingly, polyamines have been determined to prolong pyruvate dehydrogenase (PDH) activity for enhanced production of acetyl-CoA in the TCA [59]. In this study, polyamines did not necessarily upregulate PDH, but rather supported its activity. Thus, it may be extrapolated that Met use for polyamine production not only prevents pyruvate generation via transsulfuration, but also drains pyruvate pools through PDH for TCA use (Figure 4).

In addition to the aforementioned canonical Met cycle/transsulfuration/sulfur transferase-dependent production of pyruvate, recent work indicates alternate transsulfuration mechanisms by which CTH can also generate pyruvate from the oxidized form of Cys, cystine (Figure 4). Cystine is imported to the cell by the cystine-glutamate antiporter xCT. Cytosolic cystine may then be cleaved by CTH to cysteine hydropersulfide (CysSSH) and pyruvate. Though research on CysSSH biology is still immature, the potential for CysSSH to be generated in this manner may make CySSH signaling and redox biology important for the regulation of pyruvate-dependent energy production stemming from sulfur metabolism.

#### 3.1.2. α-Ketogluturate

Anaplerosis for *a*-KG occurs through two major sources: (1) citrate (through aconitase/IDH) and (2) glutamate (through ALT mediated deamidation). As mentioned above, the ratio of *a*-KG to OAA provides a sensor for the carbon flow status through the TCA [33,48,49,50]. Though citrate is not an amino acid, it can be fueled through amino acid metabolism for *a*-KG production

##### Citrate and Cysteine

Citrate is formed from the condensation of acetyl-CoA and OAA by citrate synthase (CS: irreversible), and its formation allows for the entry of carbon equivalents to the TCA from many metabolic sources. CoA (alongside pyruvate) is the precursor for acetyl-CoA, which is synthesized from pantothenate and cysteine. Thus, cysteine metabolism and the transsulfuration pathway are important for acetyl-CoA, citrate and ultimately, *a*-KG fueling. In addition, methionine and the methionine cycle must also regulate *a*-KG anaplerosis through its conversion to homocysteine in the methionine cycle and further to cysteine down the transsulfuration path.

#### 3.1.3. Pyruvate and *a*-KG

Up to this point, all the amino acid metabolism discussed has been geared toward fueling individual master nodes. However, one amino acid, serine, can dually fuel the pyruvate and *a*-KG nodes, making it distinct from most other amino acids in this regard. Thus, the following section discusses serine’s role in fueling both the pyruvate and *a*-KG nodes.

##### Serine

Serine metabolism, similar to Cys, is unique among the amino acids because it acts at both the pyruvate and *a*-KG nodes [60,61,62]. Moreover, serine-dependent pyruvate and *a*-KG production also occurs through transsulfuration processing of serine. While serine is a nonessential amino acid, its de novo synthesis is important and impacts cancer cell metabolism by diverting glycolysis and rewiring the carbon flow [63]. Biosynthesis of serine begins with the glycolysis intermediate 3-phosphoglycerate (3PG), which is diverted from glycolysis for serine synthesis. The commitment step for serine biosynthesis is oxidation of 3PG by phosphoglycerate dehydrogenase (PHGDH) to 3-hydroxyphosphopyruvate, and this enzyme is elevated in a number of cancers, including breast [64,65]. Next, 3-hydroxyphosphopyruvate is transaminated by phosphoserine transaminase (PSAT1) to 3-phosphoserine, which is finally dephosphorylated by phosphoserine phosphatase (PSPH) to yield serine [66]. Interestingly, serine inhibits PKM1/2, thereby also preventing completion of glycolysis and pyruvate production.

Aside from drawing from the fuel pool, serine is also a source of numerous biomolecules, including pyruvate. Additionally, serine can be converted to glycine by serine hydroxymethyltransferase (SHMT), and this serine-glycine exchange feeds into the one carbon/folate cycle to provide methyl equivalents to the cell [66] and also overlaps with the Met cycle by providing 5,10-methyltetrahydrofolate (5,10-mTHF) for Met assembly from HCys. Glycine’s role in collagen synthesis also links serine metabolism to collagen assembly and the ECM.

Another metabolic fate for serine is its catabolism via dehydrogenation by serine dehydratase (SDH) to 2-aminoacrylate (2-AA). Serine catabolism is essential in maintaining mitochondrial respiration [67] and, through several pathways, can produce pyruvate as well as OAA and malate. In addition, serine catabolism products feed into the TCA cycle for energy cycling. Also, 2-AA is a toxic intermediate that is further metabolized by 2-iminobutanoate/2-iminopropanoate deaminase (RidA), which deaminates 2-AA to ammonia and pyruvate as end non-toxic products [68]. Thus, in addition to pyruvate production in the transsulfuration path from serine, serine catabolism through 2-AA also yields pyruvate. Some in vitro studies show that serine deprivation is not alleviated by extracellular serine in the media, suggesting intracellular serine biosynthesis is critically important. However, hyperactivation of serine metabolism can increase oncogenesis, driving a number of essential pathways in cancer cells [66], including regulation of glucose-derived carbon flow (mentioned above), production of methyl equivalents (via 5,10-mTHF), regulation of sulfur flow through the transsulfuration path (via Met), construction of TCA components (i.e., pyruvate), folate path regulation and regulation of nitrogen flow (via glycine-derived citrulline). Conversely, serine starvation will induce p53 which leads to increased cellular protection, while p53 incompetence leads to increased cell death. This pathway is associated with a more aggressive breast cancer cell.

### 3.2. Indirect Amino Acid Fueling

#### 3.2.1. Arginine

Arginine metabolism is at the heart of cancer as well as several inflammatory diseases. While not associated with direct anaplerosis to the TCA, its impact on cell growth as well as immune function is critical. There are two major branches in arginine metabolism, NO and polyamine synthesis. In cancer cells, inducible NOS (NOS2) expression favors a mesenchymal or CSC-like phenotype [8,10]. On the other hand, polyamine production is generally associated with a more proliferative phenotype [69]. In immuno-oncology, increased arginase and polyamines are associated with immunosuppression, while in murine myeloid cells, NOS2 is considered to be proinflammatory or M1-like [69]. Additionally, NOS2 is important in signaling and intercellular communication, and NOS2-expressing immune cells can lead to the conversion of a proinflammatory to an immunosuppressive phenotype [70,71]. Thus, the context and timing are essential (Figure 5).

#### 3.2.2. Nitric Oxide

The first branch of arginine metabolism is its conversion to NO via NOS. In biological systems, the chemistry of NO, which is a radical species, primarily restricts its direct reactivity to: (1) metal complexes (i.e., heme and non-heme iron proteins) [35,69] and (2) reactive radicals (i.e., reactive oxygen species (ROS)). In combination with its innate chemical reactivity, NO-dependent cell signaling is largely dependent on steady state NO concentrations. For example, NO concentrations below 100 nM are generally considered to regulate cyclic-GMP activity and vascular tone via NO interaction with the heme protein soluble guanylyl cyclase (sGC) [19]. Nitrosative signaling, which occurs at NO fluxes of 300–500 nM (Figure 1), results from the chemical reactivity of nitrogen oxides derived from the reaction of NO with dioxygen (O_2_; a radical species as well) [20]. Such nitrosative signaling leads to the increase of numerous pro-tumorigenic signaling pathways, including activation of RAS/EGFR, TGF-β and Src, as well as stabilization of HIF-1α and NrF2 [15,72]. However, this same nitrosative signaling is also paramount in driving cellular wound healing mechanisms [19]. Above 500 nM, NO induces ‘nitrosative stress’ responses. In normal cells, nitrosative stress induces p53, which subsequently causes the down regulation of NOS2 expression and NO production. Likewise, p53 induction in CD4/CD25- cells causes IL-10 production, which also results in NOS2 down regulation. However, mutations to p53 that are often found in tumor cells, causes inhibition of NOS2 down-regulating mechanisms, allowing tumor cells to sustain NOS2 and NO production [73]. This is often associated with poor prognosis and patient outcome (Figure 5).

#### 3.2.3. Polyamines

In macrophages and other cells, increased polyamine synthesis occurs during wound healing. Under these conditions, rather than being utilized for NO production via NOS2, arginine is converted to ornithine by arginase (ARG) [35]. From here, ornithine is either recycled back to the urea cycle or proceeds to polyamine synthesis [74]. For return to the urea cycle, cytosolic ornithine is transported to the mitochondria by the mitochondrial ornithine-citrulline antiporter-1 (ORNT1), where it combines with carbamoyl phosphate through ornithine transcarbamylase (OTC) to form citrulline. Conversely, continuation to polyamine synthesis involves conversion of ornithine to putrescine by the enzyme ornithine decarboxylase (ODC), which is the rate limiting step for polyamine production. Thus, ODC has been called the “gate keeper” for polyamine biosynthesis, and increased ornithine levels generally indicate activation of this pathway (Figure 5).

Having important roles in cell proliferation, DNA stabilization, and chromatin structure, polyamines are arguably one of the most controlled substances in the cell, and rightfully so [75]. The majority of cellular polyamines are bound to RNA where changes in structure produced by binding to ribosomes, tRNA, and some mRNAs, influence protein synthesis in multiple ways, as well as the interaction of proteins for microtubule formation [76]. For example, the polyamine spermidine is a precursor for hypusine, which is post-translationally incorporated into eukaryotic initiation factor 5A isoform 1 (eIF5A). eIF5A is necessary for the prevention of ribosomal stalling in the translation of mRNAs encoding polyproline tracts and other specific amino acid combinations [77]. In cancer, the MYC oncogene influences hypusine formation by up-regulating transcription for the gene encoding the polyamine gate keeper ODC, promoting polyamine synthesis. Polyamines can also affect calcium and potassium ion flow in and out of the cell. Thus, the multifunctional roles of polyamines in ion channel regulation, chromatin structure maintenance, DNA replication, transcription, and translation, make them essential for cellular function.

**Figure 6:**
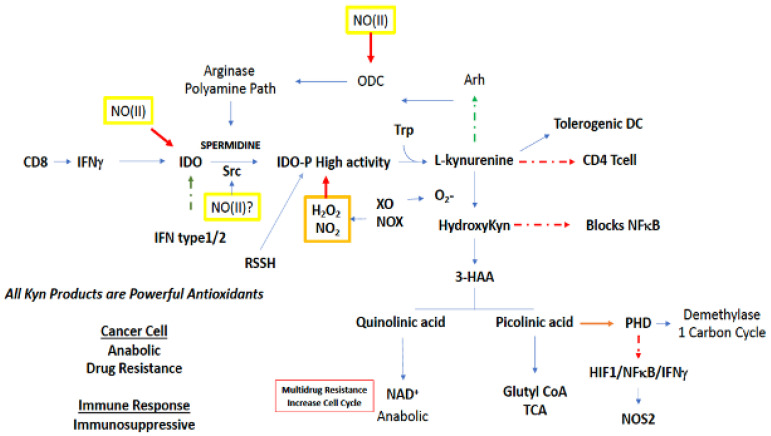
NO reactivity leading to regulation of tryptophan related metabolic pathways. Green boxes/(II) represent intermediate levels of NO (nitrosative signaling) and red boxes/(III) indicate high levels of NO (nitrosative stress) required for NO interaction at the indicated site. Red arrows indicate inhibition, green arrows indicate activation, and broken arrows designate major pathways.

Polyamine biosynthesis is generally associated with cell growth and a number of cancer-related signaling pathways have been shown to control cellular polyamine levels [74]. Increased MYC expression increases transcription for the polyamine gatekeeper ODC (Figure 6), and the RAS/ERK signaling path can increase polyamine uptake while also down regulating spermine/spermidine acetyltransferase (SSAT) [78]. The latter prevents polyamine acetylation, keeping polyamine levels high to promote cell growth. Hypoxia-induced Akt also enhances intracellular polyamine concentrations by down regulating SSAT to prevent acetylation-driven polyamine export from the cell [79]. With regard to the immune system, various polyamines drive different immune responses. For example, spermine generally drives M2 polarization, while blocking M1, whereas putrescine and spermidine increase M1 polarization while blocking M2.

In contrast to polyamine synthesis, polyamine interconversion and catabolism can promote cellular toxicity. The conversion of spermine to spermidine by spermine oxidase (SMOX) results in the production of peroxide and the aldehyde byproduct 3-aminopropanal (3-APP), both of which are toxic to the cell [31,75]. Importantly, SMOX levels are elevated in cancer, and this leads to elevated risk for both prostate and colon cancers [80]. Similar to interconversion, polyamine catabolism is also generally associated with cell toxicity. Polyamine catabolism involves acetylation of polyamines by SSAT [81]. Once acetylated, acetylpolyamines are either exported from the cell or oxidized by the peroxisomal N-acetylpolyamine oxidase (PAO), a process also resulting in peroxide and/or 3-APP. Abundant export of catabolic polyamine metabolites acetyl- and diacetylspermine/spermidine by macrophage is thought to be pro-carcinogenic through production of peroxide and 3-APP. Thus, control of enzymes associated with polyamine interconversion/catabolism is important, as outcomes may have drastic consequences.

#### 3.2.4. Agmatine

As a third metabolic route, arginine can be converted to agmatine by arginine decarboxylase (ADC), However, compared to the other two arginine metabolic paths, much less is known about its fate [82]. Agmatine itself has been proposed to activate Nrf2 (10 mM) [83] inhibit NOS2 (0.22 mM) [84], inhibit ODC, and induce spermine/spermidine acetyltransferase-1 (SSAT) to limit polyamine accumulation through induced acetylation [85,86]. Oxidation of agmatine by diamine oxidase yields an aldehyde [87], which in turn is converted by aldehyde dehydrogenase (ALDH) to guanidinobutyric acid [88]. Importantly, the latter compound has been shown to have anticlotting effects, cause convulsions and lead to acute and chronic gastric inflammation.

#### 3.2.5. Tryptophan and N-Formylkynurenine

Tryptophan is the rarest of the amino acids, and thus is of limited availability to a proliferating pathogen or tumor cell (Figure 6). Metabolic starvation is a strategy of the immune system, in particular arginine and tryptophan, to prevent the growth of pathogenic compounds. Tryptophan metabolism largely occurs in the kynurenine (Kyn) path [89,90,91]. O_2_-dependent oxidation of Trp by indoleamine 2,3-dioxygenase 1 (IDO1), which is the rate limiting step in the kynurenine metabolic path, produces N-formylkynurenine, which can decrease Th1 and Th17 activation of T cells, while also increasing T_regs_ and FOXP3, as well as the development of tolerogenic dendritic cells [89]. Furthermore, Kyn can activate Arh, which increases Tregs as well as modifies BRCA1 methylation status, p450, and increases ODC activity [92]. IDO is a very sensitive barometer of IFNγ gene expression, where its increased expression appears to be a feedback mechanism countering IFN signaling [91].

Increases in O_2_^−^ leads to the hydroxylation of Kyn, forming HO-Kyn, which inhibits development of CD4 cells. HO-Kyn is converted to 3-hydroxyanthranilic acid (3HAA), which can inhibit NFkB [91] and other proteins such as NOS2. Furthermore, 3HAA metabolites block tetrahydrobiopterin (BH_4_) synthesis though interaction with guanosine triphosphate cyclohydrolase 1 (GTPCH1), thereby inhibiting NOS2 and numerous protein synthesis as well. In addition, it causes apoptosis in Tc and Th1 cells. Kynurenine can be broken down to anthranilic acid and alanine that can be recycled [91].

3HAA can either be diverted to quinolinic or picolinic acid (Figure 6). Quinolinic acid leads to NAD^+^, which in tumor cells increases chemoresistance through PARP which is critical in DNA repair [93]. On the other hand, picolinic acid is a known metal chelator, similar to deferoxamine, that in addition to being an antioxidant, targets non-heme protein pools aconitase and prolyl hydroxylases (PHD), which in turn stabilizes HIF1α and NFκB as well as inhibits TET and Jumonji [94]. Also, as a metal chelator, picolinic acid can impact zinc metabolism. From a signaling perspective, picolinic acid, in vitro, can interact with IFNγ to induce NOS2, and this may suggest IDO within one cellular compartment can increase NOS2, via IFNγ, in an alternate compartment (Figure 6).

The interaction of the tryptophan node with other metabolic pathways occurs through cellular signaling. For example, spermidine derived from arginine/ODC interacts with Src, which results in phosphorylation of IDO, which augments its activity [95]. Thus, polyamines can augment anti-Th1 activation through IDO. While IFNγ may induce the enzyme that results in PA at a later time point, IDO represents a buffering mechanism for shutting down inflammation, and plays an important role in the coordination of immunosuppressive mechanisms. Since Kyn can increase Arh (Figure 6), which increases ODC, there appears to be a feed forward mechanism for increased polyamine synthesis. In turn, ODC leads to increased IDO expression. The primary mechanism is through IFNγ and not IL-4. Another perspective is that arginase/ODC/PA and IDO deplete arginine and tryptophan to control pathogens [89]. Meanwhile this mechanism appears to be important for the downregulation of IFNγ signaling leading to immunosuppression.

High IDO expression, like NOS2 and COX2, predicts poor outcome [90,96,97,98]. Given that IDO is a heme protein, it is susceptible to redox regulation. Peroxide, NO and NOS2 can inhibit its activity. Given the apparent antagonistic relationship of NOS2 and IDO [99], what are their roles in antitumor and antipathogen regulation? The formation of the metal chelator picolinic acid targets aconitase (citrate block) as well as PHD. Thus, NOS2 and IDO can shape the metabolic microenvironment like hypoxia or NO. They have effects on TCA/respiration and facilitate epigenetic changes. Another important aspect of IDO1 is that it increases IL-6, thereby supporting a number of pathways including angiogenesis in BrCa, which is antagonized by ERα [100]. IDO1 increases MDSC recruitment where APC cells induced by IDO1 impact the immune cellular neighborhood by blocking NK and CD8 effector cells and increasing CD4 cells [101]. IDO2 can induce autopathogenic T and B cell antibody production. Treatment with the IDO inhibitor 1-methyl tryptophan increases T-lymphocyte response, which shows that IDO is a major component of immunosuppressive mechanisms [102].

## 4. NO and Its Effect on Key Metabolic Nodes

### 4.1. Arginine

Arginine metabolism is a critical pathway for determining the inflammatory profile and targeting this pathway in numerous diseases have yielded important insights [103,104,105]. The major nexus is the partitioning between NOS2 activity and polyamine synthesis forming a metabolic node in inflammation. In principle, NOS2 is an antagonist to polyamine synthesis (Figure 6) though production of NOHA that inhibits arginase and NO direct inhibition of ODC [106]. Arginase, through its higher K_m_, can limit arginine availability, converting NOS2 to a ROS generator. In addition, the polyamine progression also increases ROS, which is a chemical antagonist to NO. This switch between NO and ROS has numerous impacts on the immune polarization in the inflammatory niche. Arginase and ODC activity are involved in many Th2 related diseases, while NOS2 is associated with M1 polarization in mice [107,108]. However, high NO levels provide anti-Th1 feedback through activation of TGFβ and induced IL-10, both of which can inhibit further NOS2 expression while favoring TGFβ immunosuppression [109,110]. NOS2 can be produced locally by a single cell level at the NO(III) level but will form a gradient with increased distance from the NOS2 expressing cell, thus having a distal effect and shaping the metabolic niche.

An example is in cancer models and tuning the immune system where the role of arginine is to promote either immune suppression through polyamine synthesis or immune activation in myeloid cells through NOS2 [111]. In aggressive tumors, arginase, and polyamines in myeloid MDSC is immune suppressive, contributing to resistance to therapy [112]. However, infiltrating immune cells such as lymphoid cells and neutrophils leads to increased cytokines that lead to conversion of tumor associated M2 macrophages (immunosuppressive) to M1 proinflammatory macrophages [113]. These macrophages generate level III NO which would be capable of inhibiting adjacent cells that produce arginase/ODC. This is a direct chemical change in the cellular neighborhood and ecosystem. The area then depresses arginase/polyamine, NO inhibits the oxygen consumption of cells and can lead to further recruitment of activated neutrophils and TILS. Thus, arginine metabolism can provide real time and spatial tuning of the immune response.

### 4.2. Tryptophan

Another complimentary system to arginine is the tryptophan oxidation to kynurenines. As discussed above, these pathways are important in immune activation or suppression. IDO is induced by the Th1 master cytokine IFNγ, which provides a check on inflammation [114]. One important factor in this pathway is that the activation of IDO through phosphorylation stimulated by polyamines dramatically increases the activity [95]. This suggests a collaboration between the arginase and IDO immunosuppressive pathways. The role of these pathways is in part to deplete critical amino acids for pathogen and tumors as a last resort. NO has a dual effect here, the first is inhibition of the polyamine preventing IDO phosphorylation. In addition, NO can directly inhibit IDO through interaction with heme [115]. An interesting aspect of this mechanism is that picolinic acid is a powerful intracellular metal chelator, which can lead to modification of PHD which regulates HIF1α and IKKα as well as epigenetic events [116]. In some cases, this can lead to increased NOS2 expression, suggesting an alternative tuning mechanism of this system [117]. Thus, the collaboration between arginine and tryptophan metabolism is antagonistic with respect polyamine versus NO synthesis. This is another example of how NO can tune the immune response through adjusting amino acid metabolic pathways.

One interesting species difference is that exposure of human macrophages to IFNγ and LPS induces COX2 and IDO, while NOS2 is induced in mouse macrophages (NO level III) [69,118,119]. This difference may reflect an evolutionary difference in the strategy used to fight pathogens in rodents vs. humans. From a chemical perspective, there are more similarities: where IFNγ and LPS induces COX2 in both species, iron chelators and antioxidants formed from the kynurenine pathway have a similar effect as does high NO levels. Iron chelators such as picolinic acid target iron sulfur and nonheme iron pools, as does NO, resulting in simulated hypoxia. NO and kynurenines are antioxidants that shift ROS type signaling as well as protect against cellular damage. From this perspective, the human and murine macrophage have similarities in cell biochemical targeting and cellular phenotypes but simply use a different messenger through different biochemistry.

### 4.3. G6PD

NO concentrations associated with nitrosative stress have been reported to stimulate G6P shuttling to the PPP in neutrophils [120]. This is because NO, at this concentration, causes protein *S*-nitrosation, which can be viewed as an oxidative-type modification to cysteine. Thus, to reverse this type of oxidative stress, the cell shuttles G6P to the PPP for NADPH production in an attempt to reduce *S*-nitrosproteins back to the original sulfhydryl through thioredoxin. These conditions will ultimately divert glucose from glycolysis to the PPP thus, limiting pyruvate for TCA use, resulting in decreased TCA-derived *a*-KG and OAA. To counter this, the cell may utilize glutamine/glutamate equivalents to make *a*-KG for alternative TCA fueling. Alternatively, in colorectal cancer cells, PPP stimulation through G6PD overexpression and subsequent NADPH allows these cells to thrive by recycling more GSH for ROS scavenging [121].

### 4.4. GAPDH

High NO concentrations associated with nitrosative stress have been shown to induce S-nitrosation of GAPDH. Specifically, LPS stimulation of RAW murine macrophage causes upregulation of iNOS and NO, which ultimately leads to GAPDH S-nitrosation [122]. This post-translational modification induces GAPDH binding to Siah1, forming a complex that translocates to the nucleus and signals apoptosis. Importantly, this inhibitory effect of NO will cause a backup of G6P, which may be shuttled to the PPP for metabolism and prevention of G6P accumulation. This will also prevent pyruvate synthesis and proper TCA cycling for energy production. Furthermore, as GAPDH lies upstream of 3PG, this will ultimately limit 3PG levels and its availability for serine synthesis and thus, those amino acids derived from serine, including glycine, arginine, Met and Cys. A lack of glycine will also augment ECM modeling, while a lack of arginine and Met will decrease polyamine synthesis and proliferation.

### 4.5. PKM/PFK

At low concentrations (≤100 nM; NO/nitrosative signaling) NO stimulates PKM2 translocation to the nucleus and induces transcription of glycolytic genes, including PFK1, resulting in increased glycolytic activity [123]. However, high nitrosative stress NO levels cause decreased glycolysis. In this manner, low levels of NO facilitate pyruvate production from glycolysis to be used in the TCA, resulting in increased *a*-KG and OAA. However, this will also prevent G6P from the PPP and 3PG from serine synthesis thus, anabolic pathways limiting glycine, arginine, Met, Cys and polyamine synthesis as well.

### 4.6. PDH/Aconitase

High levels of NO resulting from IFNγ + LPS stimulation of murine bone marrow-derived macrophage was shown to inhibit pyruvate entry to the TCA through PDH, but not PC, in a NOS2 and NO-dependent manner [28]. In this same study, NO also inhibited aconitase activity and decreased the TCA-derived metabolites glutamate and *a*-KG, while increasing extracellular glutamine uptake. Along these lines, it may be hypothesized that a lack of TCA-derived *a*-KG may also prompt the cell to pull this metabolite from other sources as well, akin to upregulation of glutamine import, as reported. Thus, in addition to the observed NO-derived metabolic reprogramming, such conditions may also increase sulfur and serine metabolism through the transsulfuration path and proline catabolism through OCD to replenish *a*-KG. Interestingly, proline catabolism also affords OAA as a byproduct, which may also feed into the TCA during an NO-derived TCA break. Thus, regarding glycolysis and the TCA, low levels of NO stimulate glycolysis while high levels of NO inhibit it (and the TCA), while stimulating G6PD and the PPP. In other words, low levels of NO will force glucose through glycolysis whereas higher levels will shunt it to the PPP.

### 4.7. POX

POX is a p53 regulated gene that was shown to produce ROS, resulting in apoptosis of ovarian cancer cells [124]. As mentioned, p53 stimulation is sensitive to NO-induced nitrosative stress, making NO a regulator for POX and POX-induced ROS and apoptosis. Thus, through POX, high NO flux may cause a shift in amino acid metabolism towards proline oxidation, utilizing metabolites like OAA and *a*-KG to sustain adequate proline for POX use.

### 4.8. PHD

Under normoxia, moderate levels of NO (100–300 nM) was shown to stabilize HIF-1α expression through the inhibition of PHD [125]. PHD is a O_2_, ascorbate and *a*-KG dependent enzyme and therefore, PHD inhibition by NO can be anticipated to cause a surplus of *a*-KG.

## 5. Conclusions

The interplay between NO, signal transduction and metabolism are intertwined and is dependent on the concentration of NO. This triad of regulation is the basis of shaping the cancer cell environment as well as in other diseases. The progression of inflammation and metabolism is a re-emerging area where there are new diagnostic and therapeutic opportunities.
